# Comparative effects of curcumin, nano curcumin and their combination on reproductive traits and spawning performance of red tilapia (*Oreochromis Niloticus* X *O. Mossambicus*)

**DOI:** 10.1186/s12917-024-04257-8

**Published:** 2024-09-21

**Authors:** El-Sayed Hemdan Eissa, Basma M. Hendam, Hagar Sedeek Dighiesh, Heba E. Abd Elnabi, Yasmin M. Abd El-Aziz, Moaheda E. H. Eissa, Sameh A. Abdelnour, Sara F. Ghanem

**Affiliations:** 1 Fish Research Centre, Faculty of Agricultural Environmental Sciences, Arish University, El-Arish, Egypt; 2 Department of Animal Wealth Development, Faculty of Veterinary Medicine, Mansoura University, Mansoura, 35516 Egypt; 3 Department of Aquaculture, Faculty of Fish Resources, Suez University, P.O. Box:43512, Suez, Egypt; 4 Department of Fish Resources and Aquaculture, Faculty of Environmental Agricultural Sciences, Arish University, El-Arish, Egypt; 5 Zoology Department, Faculty of Science, Port Said University, Port Said, 42526 Egypt; 6 Biotechnology Department, Fish Farming and Technology Institute, Suez Canal University, Ismailia, Egypt; 7 Department of Animal Production, Faculty of Agriculture, Zagazig University, Zagazig, 44511 Egypt; 8 National Institute of Oceanography and Fisheries, NIOF, Cairo, Egypt

**Keywords:** Nano-curcumin, Red tilapia, Sex hormones and reproduction, Hemato-biochemical indices, Reproduction- associated genes

## Abstract

Curcumin, the main polyphenol component of turmeric powder, has garnered increasing attention as an effective supplement in fish diets. A comparative trial was conducted to evaluate the impacts of dietary supplementation with different forms of curcumin (free, in combination, or nanoparticles) on hemato-biochemical parameters, reproductive capacity, and related gene expressions of red tilapia (*Oreochromis niloticus* x *O. mossambicus*) broodstock. Fish (*n* = 168) were fed an isonitrogenous (30% CP), isocaloric (18.72 MJ kg − 1) diet containing basal diet (Control), 60 mg kg-1 of either free curcumin (Cur), curcumin/nano-curcumin blend (Cur/NCur), or nano-curcumin (NCur) for 56 days. Red tilapia broodstock (155 ± 5.65 g) were stocked at a male: female ratio of 1:3. Blood samples and gonads were collected to assess hemato-biochemical parameters, reproductive capacity, and related gene expression at the end of the feeding trial. The results indicated that the values of hematological parameters (RBCs, WBCs, hemoglobin), total protein, albumin values, and reproductive hormones (T, LH, and FSH) were significantly increased, while liver function enzymes were decreased in the NCur group (*P* < 0.05). Reproductive performances (GSI, gonad maturation, total number of fry per female) were significantly improved in the NCur group compared with those in other groups (*p* < 0.05). The expression of reproductive genes (*CYP19A1A, FSHR, LHR, FOXL2A, ESR1, ESR2A,* and *PGR*) were significantly up‐regulated in the gonads of fish fed NCur. Collectively, feeding red tilapia diets containing NCur led to noticeably better results followed by Cur/NCur blend, then free Cur compared to the control diet. These results indicate the superiority of NCur over its free or blended form, suggesting that a diet containing about 60 mg/kg of NCur is beneficial for enhancing hemato-biochemical parameters, improving reproductive performance, and enhancing the gonadal architecture of red tilapia.

## Introduction

The upcoming needs for food in required amounts can be satisfied from aquaculture by the accessibility of affordable and high-quality feed [1–4]. Due to its fast growth rate, nutrition value and availability, hybrid red tilapia is broadly cultivated and consumed occupying a strategic position in the market [5–7]. Being similar to the red snapper, the color and body shape of this tilapia (*Oreochromis niloticus* x *Oreochromis mossambicus*) make it most profitable fish species worldwide [8, 9]. Currently, plant-based additives are broadly applied for their numerous advantageous properties in aquaculture [6, 10, 11]. Curcumin (Cur) is a yellow active molecule in the rhizomes of turmeric powder *Curcuma longa* [12]. It acts as antioxidant, anti-inflammation and immunostimulant agent [13, 14]. Moreover, inclusion of curcumin in the diets of juvenile rainbow trout (*Oncorhynchus mykiss*) [13], crucian carp (*Carassius auratus*) [15] and Nile tilapia (*Oreochromis niloticus*) [16] improved fish health status and its innate immune response [17]. Further, [18] reported a significant increase in hematological parameters and a regulation in liver functions of Gilthead seabream (*Sparus aurata*) fed 2–3% dietary curcumin. Besides, nutrition is essential to the reproductive performance of tilapia and all vertebrates. Modulation of the diet composition and the use of functional feed additives has become an extensively accepted technique to increase the performance of aquatic animals [19–21]. Generally, the reproductive axis is inhibited due to negative energy balance and low food consumption [22]. Successful reproduction involves suitable resources to sustain the high-energy required for gametogenesis and reproductive behaviors. Moreover, feed quality of fish is also important for its survival rate, hatching rate, fecundity, gonadal development, and maturation as well as reproduction performance [23]. To enrich feed quality, the study of curcumin incorporation on fish diets has been confirmed its biological activities for accelerating gonadal development and growth performance in multiple fish species such as: common carp [24, 25], Nile tilapia [26] and Green Terror [27]. However, Curcumin has several problematic delivery issues, comprising poor absorption and low bioavailability. Owing to its unstable structure, curcumin levels are low in blood and animal tissues due to its rapid metabolism and eviction from the body, which limits its usage and persistence [28]. Fagnon et al. [17] reported the poor absorption and rapid excretion of curcumin by oral absorption in rats and other species.

Recently, nano-delivery approaches in aquafeed is progressively documented and extensively applied on fish due to its effectiveness overall productivity and performance. Consequently, dietary supplementation of curcumin nanoparticles has been applied to enhance the stability, bioavailability, circulation and distribution of curcumin in its free form [29]. In support, [30] demonstrated the higher systemic bioavailability of nano-curcumin (NCur) in plasma and tissues if compared to its free form. This may be due to the zeta potential of NCur that allows its complete dissolution in water without any aggregation [31]. Elabd et al. [32] also documented the security of the better efficiency and delivery of curcumin nanoparticles which reduces its incorporated amount required to be present in fish diets. This leads to overall improvement of Cur properties at a lower cost. Nevertheless, few studies have been conducted on curcumin or its nano spheric forms to promote fish growth, metabolism, reproduction, immunomodulatory and antioxidant properties [20, 21, 31]. However, no research has been conducted to compare the impact of nano curcumin with that of its free or mixed form on red tilapia reproduction. Whether dietary NCur would affect gonadosomatic index, egg diameter, gonad maturation, and overall reproductive performance of tilapia remains to be seen. Therefore, this work aims to assess a comparative study on the effects of dietary supplementation with free or nano-form of curcumin and both together in a blend on improving the physiological function, plasma concentrations of steroid hormones, gene expression, and the development of the reproductive organs in red tilapia (*Oreochromis niloticus* x *O. mossambicus*).

## Materials and methods

### Curcumin nanoparticles (NCur) preparation

**Fig. 1:**
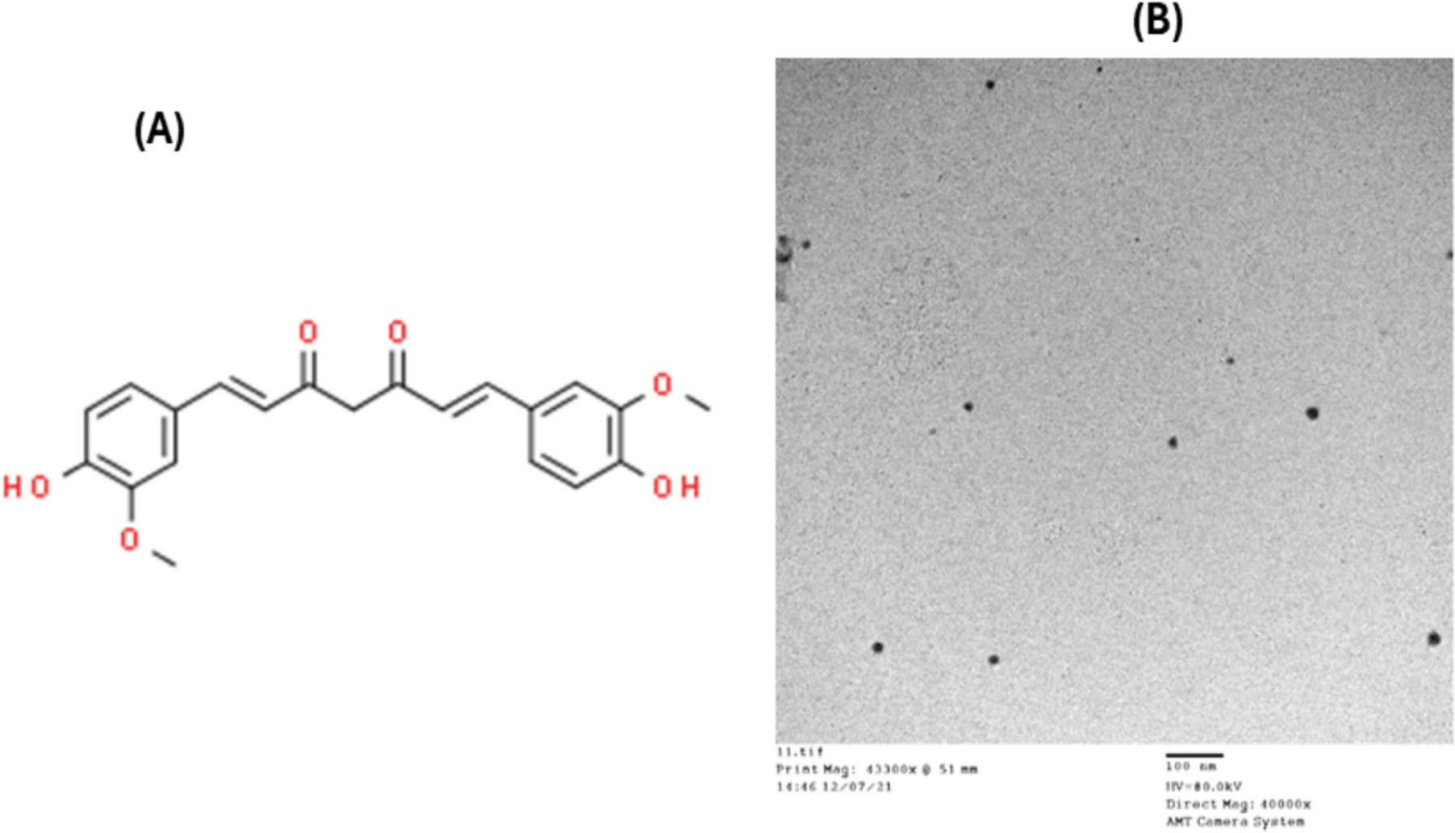
**A** and **B** The chemical structure of curcumin with the molecular formula: C_2_1H_20_O_6._(Fig. 1A). The mean size of nano-curcumin under transmission electron microscopy (Fig. 1B)

Curcumin with the molecular formula: C_2_1H_20_O_6._ (obtained from Chemajet Com., New Borg El Arab, Alexandria, Egypt) as shown in Fig. 1A, and Dichloromethane (obtained from Elgomhoreya pharmaceutical company, Zagazig, Egypt) were applied for the synthesis of curcumin nanoparticle (NCur). To synthesize nano-curcumin (NCur), the"solvent-antisolvent"method designated by [33] was used with minor modifications. Briefly, a syringe pump involving antisolvent was used to NCur manufacture employing dichloromethane as an organic solvent [34]. First of all, normal curcumin solution was prepared in dichloromethane (5 mg/mL), placed in a syringe (20 mL), and injected at a ratio of 10 mL/min into the deionized water (antisolvent), and stirred vigorously (1kg) for 2 h. Then, the vacuum-dried manufactured nanoparticles were cleaned. Assessment of NC measurement was accomplished by using Zeta sizer (Malvern Instruments, Zeta Potential Analyzer, Malvern, UK) as seen in Fig. 1B. Furthermore, the transmission electron microscope (TEM) was also applied to evaluate the size and distribution of the synthesized NCur (particle size 10–50 nm).

### Experimental design and feeding regime

The present work was conducted at the Fish Research Centre, Faculty of Environmental and Agricultural Sciences, Arish University, North Sinai, Egypt. Prior to the beginning of the trial, red tilapia (*O. niloticus* x *O. mossambicus*) acquired from the Fish Research Centre were stocked in 12 concrete ponds (3.5 × 3.2 × 0.9 m) for two weeks to acclimatize fish to indoor laboratory conditions. Then, a total of 480 red Tilapia broodstock with an average body weight of 155 ± 5.65 g were randomly allocated to forty per pond (1 male to 3 females) with 3 replicates for eight weeks. All fish individuals in each pond were weighed collectively after the acclimation period, and the average initial weight was noted. Two females were tagged by cutting small parts of the upper or lower edges of their tails, while the third female was left uncut.

The different water quality parameters were tested, recorded, and adjusted twice a week before changing the pond water throughout the experimental period using a YSI-556 multi-parameter device. Throughout the experiments, the water temperature was 26.50 ± 0.41°C, pH 7.6 ± 0.3, salinity 2.6 ± 0.31 ppt, dissolved oxygen 6.80 ± 0.41 mg/L, nitrogen dioxide 0.042 ± 0.001 ppb, and total ammonia 0.4 ± 0.01 mg/L. The permitted ranges for fish growth were met by each of these water quality metrics. Approximately 10% of the water in each tank was changed daily during the acclimation and experimental periods. Fish feces were removed, and the water was replaced with new and well-aerated water from a storage tank. Fish in each tank were weighed together every 10 days and their daily ration of food was adjusted accordingly. All relevant institutional and national policies regarding the handling and usage of animals have been applied according to the Arish University in Egypt, with Research number (Agri 06).

### Test diets

All ingredients and proximate chemical composition of the control diet comprising 30% crude protein (CP) were represented in Table 1. The dietary chemical analysis was analyzed following the AOAC protocol as described previously by [21]. The control diet was enriched with Cur, NCur or Cur/NCur blend (1:1). To make the NCur diet according to study protocol, we first mixed it with 100 mL of distilled water. We then sprayed the mixture evenly into the diet ingredients and mixed well for 30 min. Finally, we used a pelletizer to create pellets that were 1–2 mm in diameter. We stored the finished pellets in plastic bags at -4 °C for pending use. Four dietary treatments were prepared as follow: basal diet (Control), dietary supplementation with free curcumin (Cur), a blend of Cur/ NCur and nano-curcumin (NCur) corresponding to the tested levels 0, 60, 60 (30mg free Cur + 30 mg NCur) and 60 mg kg^−1^, respectively. In the present trial, the red tilapia were fed with the experimental diets at a daily rate of 3% of their live biomasses up to apparent satiety twice a day at 9:00 am and 3:00 pm. After the feeding trial, the fish tanks were cleaned and the fish were prepared for spawning. Ten ripe males and 30 ripe females (male: female ratio of 1:3) were stocked in the culture tanks and fed their assigned diets for 56 days. During this period, reproduction capacity and spawning performance were determined.

**Table 1:** The experimental diet's ingredient breakdown and chemical analysis (%, DM)

Ingredients composition (%)		Chemical analysis (%)	
Soybean meal (44% CP)	21	Crude protein (CP)	30.31
Fish meal (70% CP)	21	Crude fiber (CF)	5.31
Yellow corn	22	Ether extract (EE)	5.63
Rice bran	20	Ash	5.61
Wheat bran	10	Nitrogen free extract (NFE)	53.13
Linseed oil	2		
Minerals premix	2		
Vitamins premix^(1)^	2		

^1^Vitamin premix (per kg of premix): riboflavin, 2.5 g; pantothenic acid, 100.0 g; thiamine, 2.5 g, pyridoxine, 2.0 g; biotin, 0.3 g; inositol, 100.0 g; para-aminobenzoic acid, 2.5 g, folic acid, 0.75 g; choline, 200.0 g, cyanocobalamine, 0.005 g, nicotinic acid, 10.0 g; a-tocopherol acetate, 20.1 g, cholecalciferol, 500,000 IU, retinol palmitate, 100,000 IU; menadione, 2.0 g

### Blood sampling

The fish were anaesthetised with a 50 mg/L bath of tricaine methanesulfonate (MS-222) buffered with a 2:1 ratio of sodium bicarbonate. Blood was drawn from the fish's caudal veins after the 56-day test period. The fish in each pond were weighed, and five fish were randomly selected and given 120 mg/l amino-benzoic acid (MS-222; Sigma-Aldrich) to collect blood samples. The blood samples were collected in heparinized and clean tubes for hematological investigations. The sera were obtained by centrifugation of the blood samples at 1610 g for 10 min, and then stored at -20 °C until needed.

### Haematological and biochemical assays

Following the method defined by [35] red blood cells (RBCs) and white blood cells (WBCs) were counted using a Hemocytometer [36] method was applied to evaluate MCV (Mean corpuscular volume), MCH (Mean corpuscular Hemoglobin), MCHC (Mean corpuscular hemoglobin concentration) and hemoglobin (Hb) levels using an automated hematology analyzer (Hospitex Diagnostics, Sesto Fiorentino, Italy). According to [37] the hemoglobin was instantly assessed after being enriched with Drabkin’s solution. PCV (Packed cell volume) was evaluated by the micro hematocrit technique following [38]. Moreover, neutrophils, lymphocytes and monocytes were counted using an Olympus oil-immersion light microscope (1000X magnification). Prepared whole blood smears were checked out for phagocytic index and phagocytic activity as specified in as specified in the method of [39] method. In addition, the serum lysozyme levels were assessed following [40] protocol.

The Diamond Diagnostics Company kits were used to test serum total protein, albumin, triglycerides, creatinine, urea and uric acid using the techniques developed by [41–43] and [44], respectively [45] method were used to determine the globulin level. The colorimetric glucose oxidase method was used to measure the glucose concentrations [46]. Serum cholesterol was determined using the methods of [47]. An automated clinical analyzer (Abbott Alcyon 300, USA) was used to assess the levels of aspartate aminotransferase (AST), alanine aminotransferase (ALT) [14], and alkaline phosphatase (ALP) based on to the commercial kits provided by (Hipro Com., Shijiazhuang, China).

### Digestive enzymes activities and hormones assessment

Fish-specific diagnostic reagent kits (Cusabio Biotech Wuhan, China) were used to determine the activities of fish digestive enzymes in sera following the manufacturer’s guidelines. Serum amylase and lipase activities were assessed following the methods of [48] and [49], respectively. According to [50] commercial ELISA test kits with the catalogue numbers BC-1113 (BioCheck, Inc.) was used to evaluate reproductive hormones comprising testosterone, luteinizing hormone, follicle-stimulating hormone, 17β-estradiol and progesterone levels in blood serum.

### Organo-somatic indices

At the completion of the experiment, the total body length and weight of every male and female broodstock fish from each pond were recorded. To calculate the gonadosomatic, hepatosomatic and viscerosomatic indices, the gonads, liver, and gut were weighed, respectively. According to [51], the gonadosomatic index (GSI) was computed as:

$$\text{GSI }=\text{ gonads weight }(\text{g})/\text{body weight }(\text{g}) \times 100.$$

$$\text{Hepatosomatic index }(\text{HSI \%}) =\text{ liver weight }(\text{g})/\text{body weight }(\text{g}) \times 100.$$

$$\text{Viscerosomatic index }(\text{VSI \%}) =\text{ visceral weight }(\text{g})/\text{ body weight }(\text{g}) \times 100.$$

### Egg collection and measurements

In the present study, all female fish specimens were selected in the spawning season. By visual examination, all females were observed spawned in each pond carrying eggs in their mouths after two weeks of the experiment. The fish eggs were then collected from each female during their spawning season. After egg collection, each female was returned back to her specific pond until the end of the experiment. It was assumed that all eggs were obtained at the same developmental stage because they were stripped straight from gravid females and immediately stored in 10% formalin [52].To find the average egg diameter, a subsample of 10 randomly chosen eggs from each fish were measured. The fish eggs were counted with an insulin syringe fitted with a 1 mm needle. The females in each tank were checked daily for eggs or fry. Eggs remained in the females’ mouths until they hatched, and the yolk sac was absorbed. Hatched fry were then collected from each female, counted and their average weights were computed according to [53]. The average number of fries per spawning is calculated by dividing the total number of fries in a pond by the number of the spawned females.

### Histological examination of gonads

At the end of the feeding trial, the effect of curcumin dietary supplementation on the histological integrity of red tilapia testicular and ovarian tissues was examined according to [54]. Specimens were fixed in 10% neutral buffered formalin for 48 h, embedded in paraffin blocks and cut by rotatory microtome (5-7 µm). Subsequently, histological sections were stained with haematoxylin and Eosin (H&E) and examined with light microscopy.

### cDNA production and total RNA extraction

Red tilapia testis and ovary samples were frozen in liquid nitrogen to study gene expression. Using the Trizol reagent (iNtRON Biotechnology, Inc., South Korea), total RNA was extracted from 50 mg of ovarian and testicular tissues to examine the expression of genes involved to reproduction. Nanodrop (Uv–Vis spectrophotometer Q5000/Quawell, Quawell Technology, Inc., San Jose, CA, USA) verified the concentration of the isolated RNA. According to the manufacturer's instructions, the complementary DNA (cDNA) was synthesized using the Fast Hisenscript TM RH (Bioline, United Kingdom) RT PreMix cDNA synthesis kit from iNtRON Biotechnology, Inc., South Korea. Then, cDNA samples were stored until usage at -20 °C.

### Real time qPCR (RT-PCR)

Specific primer sequences of reproductive associated genes such as *CYP19A1A*, *FSHR*, LHR, *FOXL2A*, *ESR1, ESR2A* and *pgr* for both testicular and ovarian tissues were used. The product size and their NCBI GenBank accession numbers were depicted in Table 2. Additionally, the housekeeping gene beta-actin (*β-actin*) was employed to normalize the mRNA expression of these genes. RT-PCR was implemented using SYBR Green PCR Master Mix for the quantification of the mRNA expression folds of the target genes (SensiFast™ SYBR Lo-Rox kit, Bioline). The thermocycling conditions were 95 °C for 10 min, followed by 40 cycles at 94 °C for 15 s, 60 °C for 1 min, and finally 72 °C for 20 s. The mRNA expression folds of each target gene were standardized and normalized using the 2 ^− ΔΔCT^ method [55]. SYBR Green PCR Master Mix was used to apply RT-PCR in order to quantify the mRNA expression folds of the target genes (Sensi Fast™ SYBR Lo-Rox kit, Bioline). Thermocycling was done under the following conditions: 95 °C for 10 min, 40 cycles at 94 °C for 15 s, 60 °C for 1 min, and then 72 °C for 20 s. The 2 − ΔΔCT approach was used to standardize and normalize the mRNA expression folds of each target gene to β-actin mRNA transcripts [55]. As shown in Table 2, the product size and their NCBI Gene Bank accession numbers are listed.

**Table 2:** Primer sequences of targeted genes in hybrid tilapia applied for q-PCR analysis

Gene	Primer sequence	Genbank accessation no.	PCR product size (bp)
*CYP19A1A*	F:3'-GCATAGGCACAGCCAGCAAC-5' R:3'-GTGCACTGCTGAAGATCTGCTTAGTA-5'	NM_001279586.1	107
*FSHR*	F:3'-CGCCAGTGAGCTGTCAGTGTTT-5' R:3'-ACAGACCACGTAGAACTGGGAGAC-5'	NM_001279588.1	247
*LHR*	F:3'-ACAAGCTGACAGTACCTCGC-5' R:3'-CTGACAGCTCCCCACCAAAA-5'	XM_025897262.1	195
*FOXL2A*	F:3'-AAGAGGAGCCGGTTCAGGACAA-5' R:3'-GCTCTCCCGGATAGCCATGG-5'	NM_001279778.1	101
*ESR1*	F: 3'-ATTTTGCTAAACTCTGGTGCCTTT-5' R: 3'-GGGCTCCATTGTGCCAGT-5'	NM_001279770.1	95
*ESR2A*	F: 3'-TCCTCAACTCCAACATGTGCC-5' R: 3'-GCAGGTCCTCGCTGCAGT-5'	XM_005474955.4	120
*PGR*	F: 3'-TAGCCAGGAGCAAATGAGGAGA-5' R: 3'-TGGTAGAACCGCTGGGAACAT-5'	XM_005455073.4	264
*B-ACTIN*	F: 3'-CAGCAAGCAGGAGTACGATGAG -5' R: 3'- TGTGTGGTGTGTGGTTGTTTTG-5'	XM_003455949.2	136

*CYP19A1A* Cytochrome P450 family 19 subfamily A member 1, *FSHR* Follicle-stimulating hormone receptor, *LHR* Luteinizing hormone receptor, *FOXL2A* Forkhead box L2, *esr1*estrogen receptor 1, *ESR2A* estrogen receptor 2a, *PGR* Progesterone receptor

### Statistics

The collected results were subjected to one-way ANOVA to evaluate the effects of different forms of dietary curcumin supplementation. The differences among treatments were compared by Duncan's test using SPSS program, version 26.0. Differences were considered significant at *P* < 0.05. All results were expressed as means ± standard error.

## Results

### Haematological parameters

Table 3 shows the effects of different dietary forms of Cur on hematological parameters. The RBC counts, Hb concentration, PCV (%), MCV, and MCHC of the fish fed with different forms of Cur diet were significantly higher (*P* < 0.05) than those of the control group.The group of fish fed with 60 mg NCur kg-1 diet exhibited the highest levels of these parameters, followed by NCur/Cur group, then Cur group. However, MCHC levels, neutrophils (%), and phagocytic index did not show significant differences among all investigated groups (*P* > 0.05). WBC counts showed a slight significant increase in treated fish compared to the control group. Lymphocytes (%) exhibited a slight significant decrease in fish groups fed with NCur forms compared to the control group (*P* < 0.05). Additionally, monocytes (%) in the fish treated groups showed a significant increase compared to those in the control group. Fish in the NCur group showed significantly higher values of phagocytic and lysozyme activities compared to the control group (Table 3).

**Table 3:** Hematological parameters and immune response of red tilapia fed on different forms of curcumin supplemented diets

Parameters^1^	Curcumin Dietary Forms ( 60 mg kg^−1^)^2^			
	Control	Free Cur	Cur/NCur	NCur
RBCs (10^6^ mm^−3^)	1.60 ± 0.00^c^	1.65 ± 0.01^bc^	1.68 ± 0.02^b^	1.75 ± 0.03^a^
Hb (g dl^−1^)	7.00 ± 0.17^d^	7.53 ± 0.12^c^	8.02 ± 0.08^b^	8.58 ± 0.11^a^
PCV (%)	30.56 ± 0.39^d^	32.69 ± 0.10^c^	33.79 ± 0.13^b^	36.34 ± 0.24^a^
MCV (fl)	190.10 ± 2.44^c^	198.11 ± 1.46^bc^	200.79 ± 2.78^ab^	207.71 ± 1.86^a^
MCH (pg)	43.77 ± 1.03^c^	45.63 ± 0.61^bc^	47.65 ± 0.83^ab^	49.08 ± 1.35^a^
MCHC (%)	22.91 ± 0.31	23.03 ± 0.41	23.73 ± 0.27	23.62 ± 0.43
WBCs (× 103 mm^−3^)	40.43 ± 0.34^b^	43.85 ± 0.26^a^	43.04 ± 0.20^a^	44.67 ± 0.86^a^
Neutrophils (%)	1.58 ± 0.04	1.63 ± 0.04	1.73 ± 0.07	1.80 ± 0.10
Lymphocytes (%)	89.28 ± 0.32^a^	88.32 ± 0.16^a^	86.08 ± 0.40^b^	85.23 ± 0.51^b^
Monocytes (%)	7.33 ± 0.74^c^	9.33 ± 0.17^b^	10.23 ± 0.44^ab^	11.03 ± 0.22^a^
Phagocytic activity (µg ml^−1^)	6.40 ± 0.22^c^	8.07 ± 0.25^b^	8.53 ± 0.22^b^	9.72 ± 0.45^a^
Phagocytic index (%)	1.13 ± 0.04	1.23 ± 0.04	1.17 ± 0.09	1.33 ± 0.06
Lysozyme activity (µg ml^−1^)	1.06 ± 0.09^b^	1.14 ± 0.06^b^	1.29 ± 0.04^b^	1.52 ± 0.07^a^

^1^*RBCs* Red blood cells, *PCV* Packed cell volume, *WBCs* White blood cells, *MCV* Mean Mean Corpuscular Volume, *MCHC* Mean corpuscular hemoglobin concentration, *MCH* Mean corpuscular Hemoglobin, Values in the same row with different superscripts are significantly different (*p* < 0.05)

^2^Fish were fed basal diet (control group), or diet containing 60 mg kg^−1^ of either free curcumin (Cur), curcumin/ nano-curcumin blend (Cur/NCur) and nano-curcumin (NCur). Resuslts are expressed as Means ± SE

^a^^−^^d ^Means within a row without a common superscript letter differ at *p* < 0.05

### Biochemical analysis and digestive enzymes

The results of the biochemical blood analysis of red tilapia fed the dietary Cur forms are presented in Table 4. There was a significant effect of the experimental diets on the biochemical blood parameters of the hybrid fish (*P* < 0.05). Total protein, albumin, and triglycerides levels in fish groups fed diets supplemented with either form of NCur were higher than in the other treatments (*P* < 0.05). However, the globulin concentration was slightly increased among the different experimental groups compared to that of the control group.Liver function enzymes, ALT, AST, and ALP were significantly affected by supplemental Cur forms (*P* < 0.05; Table 3). The values of ALT and AST showed a highly significantly decreased (*P* < 0.05) in fish group fed free NCur up to 60 mg kg^−1^ diet. A similar trend has also been reported with regard to glucose, cholesterol, blood creatinine, urea, and uric acid (Table 4). Concerning the analysis of digestive enzymes, a diet of 60 mg NCur kg^−1^ displayed the highest significant lipase enzyme activity values. However, amylase activity was improved in fish groups supplemented with both forms of NCur diets (*P* < 0.05).

**Table 4:** Changes in biochemical parameters and digestive enzymes activities in red tilapia fed with various forms of curcumin supplementation

Parameters^1^	Curcumin Dietary Forms ( 60 mg kg^−1^)^2^			
	Control	Free Cur	Cur/NCur	NCur
Total Protein (mg ml^−1^)	4.21 ± 0.08^c^	4.87 ± 0.22^b^	5.55 ± 0.08^a^	5.76 ± 0.14^a^
Albumin (mg ml^−1^)	2.17 ± 0.02^b^	2.43 ± 0.26^b^	2.95 ± 0.07^a^	3.10 ± 0.08^a^
Globulin (mg ml^−1^)	2.04 ± 0.07^b^	2.44 ± 0.07^a^	2.60 ± 0.07^a^	2.67 ± 0.06^a^
Glucose (mg dL^−1^)	117.31 ± 1.41^a^	108.93 ± 0.95^b^	106.01 ± 1.31^b^	101.38 ± 1.69^c^
Creatinine (mg dL^−1^)	0.44 ± 0.00^a^	0.42 ± 0.00^ab^	0.41 ± 0.00^bc^	0.40 ± 0.01^c^
Urea (mg dL^−1^)	22.31 ± 0.51^a^	20.84 ± 0.12^bc^	21.22 ± 0.08^b^	20.19 ± 0.08^c^
Uric acid (mg dl^−1^)	1.30 ± 0.07^a^	1.24 ± 0.06^a^	1.14 ± 0.09^ab^	0.95 ± 0.05^b^
Cholesterol (mg dL^−1^)	232.53 ± 4.07^a^	194.87 ± 1.40^b^	203.27 ± 3.06^b^	168.33 ± 2.80^c^
Triglycerides (mg dL^−1^)	141.17 ± 1.96^c^	164.00 ± 5.20^b^	198.67 ± 3.06^a^	208.50 ± 3.69^a^
ALT (U/L^−1^)	53.48 ± 0.09^a^	51.86 ± 0.01^b^	50.73 ± 0.02^c^	50.15 ± 0.01^d^
AST (U/L^−1^)	147.33 ± 0.08^a^	141.72 ± 0.01^b^	138.91 ± 0.03^c^	136.65 ± 0.04^d^
ALP (U/L^−1^)	30.16 ± 0.78^a^	26.17 ± 0.23^b^	25.50 ± 0.32^b^	24.73 ± 0.23^b^
Amylase (U/L)	50.38 ± 0.97^c^	56.13 ± 0.79^b^	59.52 ± 0.50^a^	60.70 ± 0.45^a^
Lipase (U/L)	87.70 ± 0.21^d^	88.83 ± 0.18^c^	89.80 ± 0.23^b^	92.10 ± 0.17^a^

^1^Values marked with superscript letters are significantly different (*p* < 0.05)

^2^Fish were fed basal diet (control group), or diet containing 60 mg kg^−1^ of either free curcumin (Cur), curcumin/ nano-curcumin blend (Cur/NCur) and nano-curcumin (NCur). Resuslts are expressed as Means ± SE

^a^^−^^d ^Means within a row without a common superscript letter differ at *p* < 0.05

### Serum steroids

The blood levels of testosterone (T) and luteinizing hormone (LH) in male fish, follicle-stimulating hormone [56], estradiol (E_2_) and progesterone (Prog) in female fish of all groups fed with different forms of Cur were significantly increased (*P* < 0.05) compared to those in the control group (Table 5). The reproductive hormones were highest in the NCur group, followed by NCur/Cur, and then the control group (Table 5).

**Table 5:** Impact of different forms of dietary curcumin supplementation on reproductive hormones levels of red tilapia broodstock

Sex	Hormone^1^	Curcumin Dietary Forms ( 60 mg kg^−1^)^2^			
		Control	Free Cur	Cur/NCur	NCur
Male	T (pg ml^−1^)	166.67 ± 4.41^d^	222.33 ± 4.33^c^	240.00 ± 2.89^b^	258.00 ± 1.53^a^
Female	LH (mIU ml^−1^)	8.13 ± 0.03^c^	8.27 ± 0.15^c^	8.70 ± 0.12^b^	9.57 ± 0.09^a^
	FSH (mIU ml^−1^)	2.21 ± 0.01^d^	2.36 ± 0.00^c^	3.27 ± 0.00^b^	4.31 ± 0.01^a^
	E2 (pg ml^−1^)	2542.67 ± 16.51^d^	3003.33 ± 3.33^c^	3173.33 ± 37.12^b^	3311.33 ± 57.34^a^
	Prog (ng ml^−1^)	10.14 ± 0.07^d^	11.37 ± 0.32^c^	12.05 ± 0.01^b^	13.02 ± 0.01^a^

*T* Testosterone, *FSH* Follicle-stimulating hormone, *LH* Luteinizing hormone, *E2* Estradiol, *Prog* Progesterone

^1^Values in the same row with different superscripts are significantly different (*p* < 0.05)

^2^Fish were fed basal diet (control group), or diet containing 60 mg kg^−1^ of either free curcumin (Cur), curcumin/ nano-curcumin blend (Cur/NCur) and nano-curcumin (NCur). Results are expressed as Means ± SE

^a^^−^^d ^Means within a row without a common superscript letter differ at *p* < 0.05

### Reproductive performance

The findings revealed that dietary forms of Cur improved the organo-somatic indices and reproductive performance of red tilapia (Table 6). Different Cur supplements enhanced gonadal development in these fish compared to the control group (*P* < 0.05). The GSI ranged from 3.55% to 4.85% in males and 4.27% to 5.80% in females. It significantly increased in the NCur group compared to the control. Supplemental NCur and Cur/NCur blend also significantly increased the HSI and VSI in females and males, respectively (*P* < 0.05), compared to the control group. In terms of spawning efficiency and larval production, egg diameter was significantly affected by NCur supplementation (Table 6). Additionally, positive correlations (*P* < 0.05) were found between dietary curcumin forms and both the number of fry per female and fry weight (mg fish^−1^) (*P* < 0.05).

**Table 6:** Organosomatic indices and reproductive performance of red tilapia broodstock fed on different forms of curcumin supplemented diets

Parameters^1^		Curcumin Dietary Forms ( 60 mg kg^−1^)^2^			
		Control	Free Cur	Cur/NCur	NCur
**GSI (%)**	Male	3.07 ± 0.04^d^	3.55 ± 0.06^c^	4.25 ± 0.06^b^	4.85 ± 0.04^a^
	Female	4.27 ± 0.12^d^	4.67 ± 0.09^c^	5.40 ± 0.06^b^	5.80 ± 0.06^a^
**HSI (%)**	Male	2.06 ± 0.03^d^	2.36 ± 0.05^c^	2.70 ± 0.04^b^	2.93 ± 0.03^a^
	Female	1.97 ± 0.23^b^	2.07 ± 0.07^b^	2.50 ± 0.06^a^	2.67 ± 0.09^a^
**VSI (%)**	Male	10.06 ± 0.04^d^	10.95 ± 0.04^c^	11.63 ± 0.10^b^	12.05 ± 0.04^a^
	Female	13.30 ± 0.12^c^	14.10 ± 0.06^b^	14.10 ± 0.06^b^	14.67 ± 0.15^a^
**Egg diameter (mm)**		19.70 ± 0.35^d^	20.57 ± 0.12^c^	21.73 ± 0.12^b^	22.80 ± 0.15^a^
**Mean Number of fry/ fish**		777.67 ± 14.41^b^	798.33 ± 4.41^b^	952.00 ± 13.31^a^	977.33 ± 5.55^a^
**Mean fry weight (mg)**		10.43 ± 0.23^c^	11.27 ± 0.15^b^	12.47 ± 0.20^a^	13.00 ± 0.12^a^

^1^Values in the same row with different superscripts are significantly different (*p* < 0.05). GSI, Gonadosomatic index; HSI, hepatosomatic index and VSI, viscerosomatic index

^2^Fish were fed basal diet (control group), or diet containing 60 mg kg^−1^ of either free curcumin (Cur), curcumin/ nano-curcumin blend (Cur/NCur) and nano-curcumin (NCur). Resuslts are expressed as Means ± SE

^a^^−^^d ^Means within a row without a common superscript letter differ at *p* < 0.05

### Histological investigations of gonads

**Fig. 2:**
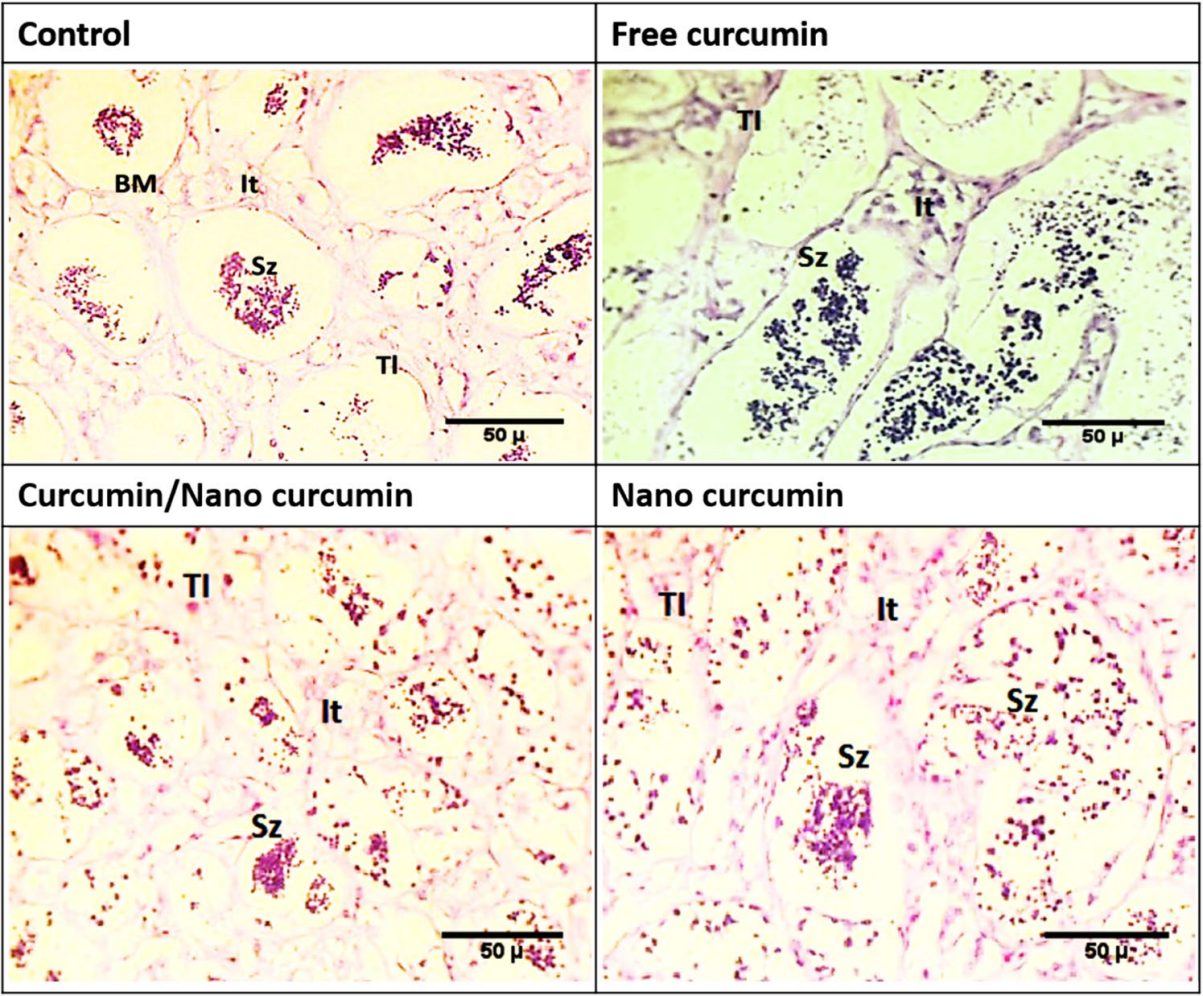
Photomicrographs of transverse sections of mature testis of red tilapia fed different forms of dietary curcumin for 56 days. Spermatozoa (Sz), testicular lobules (Tl), basement membrane (BM) and interstitial tissues (It). [H & E stain was used]. Fish were fed basal diet ( control group) or diet containing 60 mg kg^−1^ of either free curcumin, curcumin/ nano-curcumin blend and nano-curcumin

**Fig. 3:**
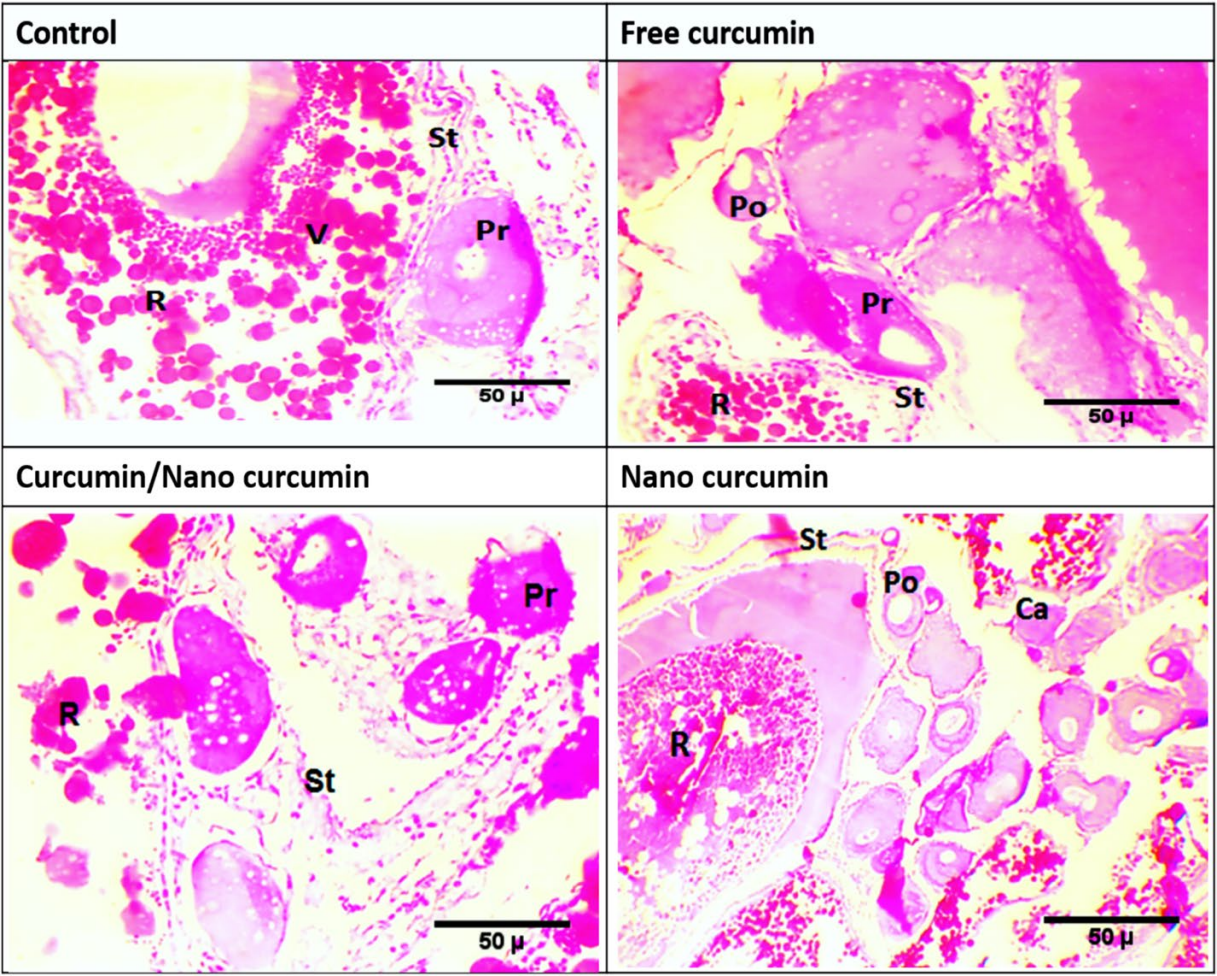
Photomicrographs of transverse sections of mature ovaries of hybrid red tilapia fed different forms of dietary curcumin for 56 days. previtellogenic (Pr) oocyte, postvitellogenic (Po) oocytes. cortical alveoli (CA), vitellogenic (V) oocyte, ripe (R) oocyte, stroma (ST). [H & E stain was used]. Fish were fed basal diet (control group),or diet containing 60 mg kg^−1^ of either free curcumin, curcumin/ nano-curcumin blend and nano-curcumin

No morphological abnormalities or surface injuries were observed in the fish samples fed different forms of curcumin diets during visual inspection in this study. The testes and ovaries of red tilapia fed the control diet showed normal tissue architecture in histological sections. Histological analysis of fish gonads from all treatment groups indicated development over the 8-week experiment. Variations in the histological profiles of male and female gonads in each experimental red tilapia group are depicted in Figs. 2 and 3.

#### Testicular changes

The histological sections of red tilapia testis in the control group demonstrate the typical architecture of seminiferous tubules with spermatogenic stages distributed inside the testicular lobules. The basement membrane is shown as a part of the seminiferous tubules wall. Further, interstitial tissues are also seen within some testicular lobules (Fig. 2a). A slight increase in the clusters of spermatozoa is detected among testicular lobules of fish group fed normal Cur (Fig. 2b). A moderate increase in the spermatozoa clusters as well as growing testicular tubules, are mainly noted in the fish group fed Cur/ NCur blend (Fig. 2c). Moreover, a marked enhancement of mature spermatozoa is observed to fill the lumen of seminiferous tubules in the fish group fed 60 mg NCur kg^−1^ (Fig. 2d).

#### Ovaries changes

The current histological preparations revealed the presence of different developmental stages of oocytes in one ovary informing that the experimental red tilapia have partial or asynchronous spawning. The histological examination of ovaries from hybrid red tilapia broodstock in the control group displayed a typical ovarian structure, which contained mature oocytes, previtellogenic, and vitellogenic oocytes surrounded by stroma (Fig. 3a). A slight increase in growing oocytes were observed in fish group fed normal Cur (Fig. 3b). This increase was more pronounced in the fish group fed Cur/NCur blend with more previtellogenic and postvitellogenenic oocytes as well as more yolk deposition in the vitellogenic oocytes (Fig. 3c). Moreover, an improvement in oocytes with post-ovulation luteinisation as well as a higher incidence of oogonia and oocytes in different developmental stages (cortical alveoli, postvitellogenenic and ripe oocytes was noted in the NCur fish group compared with the untreated red tilapia group (Fig. 3d).

### Reproduction-associated genes

**Fig. 4:**
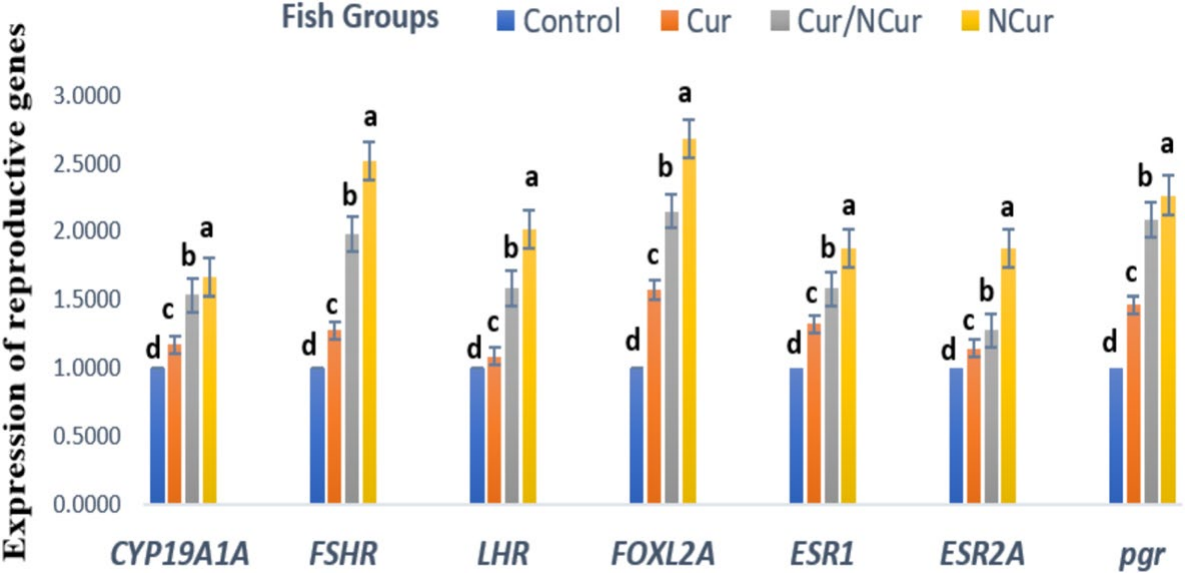
Expression of reproduction-associated genes in the testes of red tilapia fed on different forms of Curcumin supplemented diets. Fish were fed basal diet containing 0 (T0; as a control group), 60 mg kg^−1^ of either free curcumin (Cur), curcumin/ nano-curcumin blend (Cur/NCur) and nano-curcumin (NCur). Resuslts are expressed as Means ± SE. ^a−d^Means within a row without a common superscript letter differ at *P* < 0.05

**Fig. 5:**
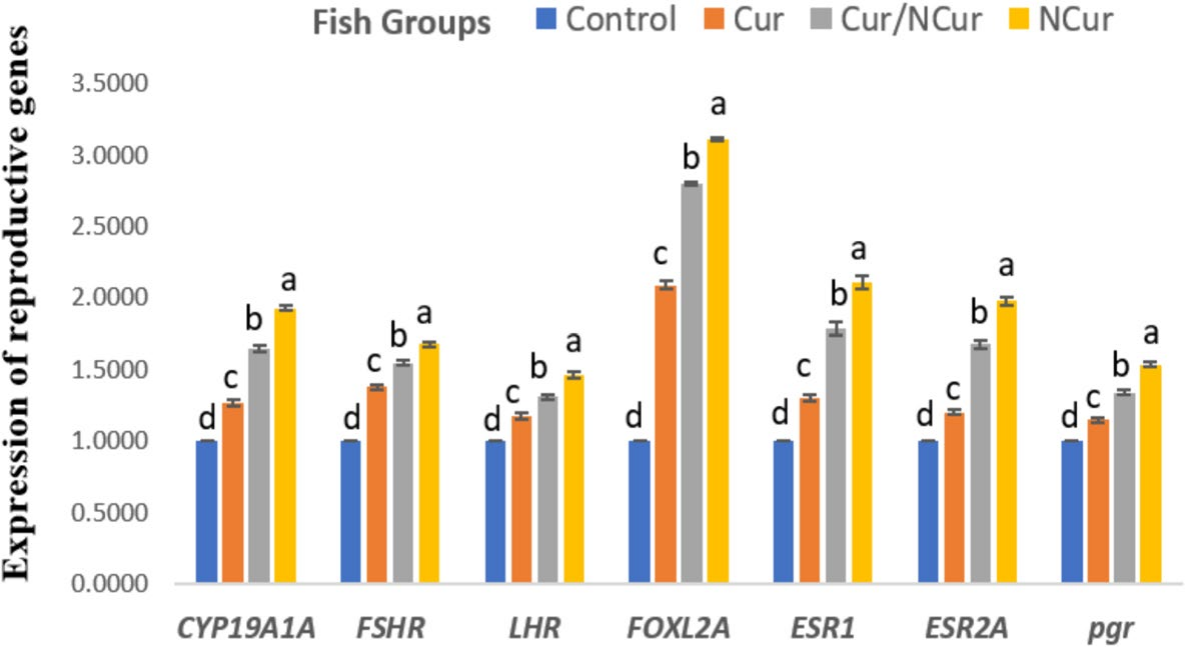
Expression of reproduction-associated genes in the ovaries of red tilapia fed on different forms of Curcumin supplemented diets. Fish were fed basal diet (Control group), 60 mg kg^−1^ of either free curcumin (Cur), curcumin/ nano -curcumin blend (Cur/NCur) and nano-curcumin (NCur). Resuslts are expressed as Means ± SE.^a−d^Means within a row without a common superscript letter differ at *P* < 0.05

The present results demonstrate that the expression of reproduction-associated genes, namely *CYP19A1A*, *FSHR,LHR*, *FOXL2A,ESR1,ESR2A and pgr* in the testicular tissue of Red tilapia was significantly affected by different dietary forms of Cur supplementation (*P* < 0.05), compared to the control group. The upregulation of these genes was superior and significantly enhanced (*P* < 0.05) in the NCur group up to 60 mg kg^−1^ diet (Fig. 4). Similarly, the expression of *CYP19A1A*, *FSHR,LHR*, *FOXL2A,ESR1,ESR2A and pgr* genes in the ovarian tissue followed the same pattern (Fig. 5).

## Discussion

**Fig. 6:**
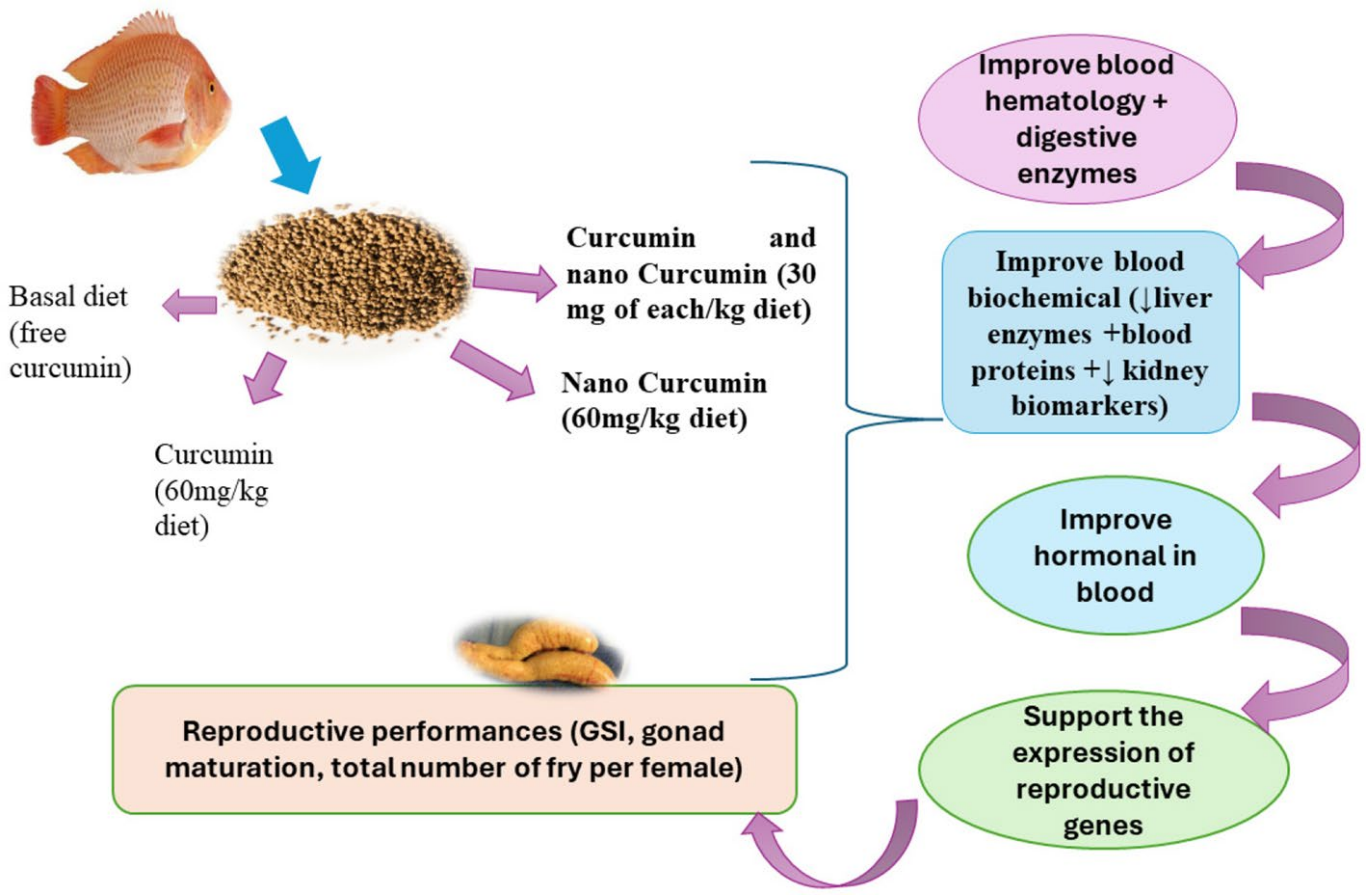
The mode of action of nano-curcumin presented in this study is to improve the blood hematology, enhance blood health (by decreasing liver and kidney damage biomarkers, as well as supporting the synthesis of blood proteins). Moreover, nanocurcumin can increase the release of blood hormones in male and female red tilapias, which supports the expression of reproductive genes. This leads to improved reproductive capacity in both female and male red tilapia broodstock

This study is the first to evaluate the effects of different dietary forms of curcumin (Cur) on gonadal development, reproductive physiology and expression of reproduction-associated genes in farmed tilapia and other fish species. Currently, the incorporation of herbal supplementation like curcumin in fish diet at low inclusion levels has demonstrated its effective role in increasing physiological, immunological functions, and the health status of fish as evidenced in this current research (Fig. 6). The hematological aspect of fish can be represented by its physiological response [16]. Generally, blood parameters in the current study are within the healthy ranges for red tilapia [57, 58]. In the current study, all blood parameters, except MCHC, neutrophils (%) and phagocytic index were significantly enhanced in red tilapia fed on different forms of curcumin supplemented diets especially in the fish group fed 60 mg NCur kg^−1^ compared to control group. This may indicate the advantage of NCur over free Cur in enhancing the heamatological parameters. The present data are consistent with the previous studies of [21] in red tilapia and [31] in Nile tilapia fed free or nano Cur. Another stuyd by [16] also revealed that a dose of 2.4 mg Cur/100 g improved the hematological parameters (RBCs, hemoglobin level, hematocrit value, WBCs and phagocytic index) of red tilapia, indicating that the use of Cur in fish diet had no adverse effect on its physiological responses. However, [27] cited that the assessed hematological parameters of the ornamental fish (*Andinocara rivulatus*) were altered by applying 0.3% turmeric powder containing curcumin to the basal diet with an emphasis on the count of WBC.

The present results also indicated that supplemental NCur led to higher levels of serum protein, albumin, globulin and triglycerides compard to other treatments. This increase may be attributed to the immune-stimulant role and antioxidant property of NCur [21]. Moreover, the increase in total proteins, globulin and albumin may be associated with the enhanced innate immune response mechanism of fish as represented by high lysozyme activity results [59]. In the present study, the increase in WBCs, monocyte percentage, phagocytic activity and lysozyme activity in the NCur treated fish group point to its role in the activation of fish immune system. The current results are consistent with previous studies [32] who suggested that the use of Cur as food supplement enhances the immune defense mechanisms of Nile tilapia (*O. Niloticus)*. The special phenolic structure of Cur is responsible for its antioxidant, immunostimulatory and anti-inflammatory properties [60].

In the present results, the results show a decrease in cholesterol, glucose, creatinine, urea, uric acid, and liver function enzymes (ALT, AST, and ALP) in the fish groups that were supplemented with curcumin, with the lowest levels observed in the NCur group. This decrease could be attributed to the hepatoprotective effect of curcumin, which helps maintain the stability of hepatocyte membranes and prevent the leakage of intracellular enzymes [61, 62]. A study by [31] also reported similar reductions in these parameters in tilapia diets supplemented with curcumin. This dual-action potential of curcumin as an antioxidant may contribute to its beneficial effects [63].

The hepatosomatic index (HSI) is a measure of the physiological, nutritional state, and overall well-being of fish [64, 65]. The present data revealed that red tilapia fed NCur exhibited improved digestive enzyme activity. Digestive enzymes are considered key constituents in the process of fish digestion [17]. The current results show that the incorporation of Cur/NCur enhanced the digestion process, as indicated by the increased amylase and lipase activities with the highest significant activity (*P* < 0.05) in the group supplemented with 60 mg NCur kg ^−1^compared to the control group.

Moreover, A study by [59] reported that curcumin can increase the enzymes activities responsible for nutrient assimilation and degradation in the gut brush border. This in turn improve digestibility, nutrient availability and utilization by decreasing nutrient excretion [15]. Moreover, the present data consistent with that of [66] who found that curcumin can efficiently raise protease, lipase, and amylase activities in the intestine of *Oreochromis mossambicus* fed 0.5% and 1% curcumin supplemented diet. This indicates that curcumin represents a good prebiotic digestive stimulant which promotes enzymatic activities and gut flora of fish improving its overall health status [26]. Similar findings were reported in goldfish (*Carassius auratus*) by [15]. These data reveal the biosafety utilization of NCur compared to its usage in free-form as confirmed by [67].

The highest male and female organosomatic indices (GSI, HSI, VSI)) were observed in the Cur/NCur and free Cur groups. Curcumin acts as a scavenger for hydroxyl radicals which in turn prevents the oxidation of biomolecules [68]. It can also improve the cells synthesis capabilities in fish bodies by playing a genoprotective role against DNA damage [69]. These reported properties of curcumin could enhance the gonadosomatic indices and hepatic function, thereby enhancing fish reproductive performance [24, 25]. Furthermore, some authors [24], have explained that the increase in liver weight, leading to an increase in HSI, may be attributed to a response to the stimulation of 17β-Estradiol, as the liver is the site of vitellogenin synthesis. Similarly, the increased gonadosomatic index observed in this study indicates an increase in gonad weight as the end product of vitellogenesis. The increased synthesis of vitellogenin recruits a higher number of developing and growing oocytes, reflected in the increase in egg diameter size and mass, ultimately leading to an increase in gonad weight and GSI [70]. Similar results were found in common carp [24, 60] and striped catfish [71], fed diets containing curcumin showing significantly better GSI, HSI and VSI than the control groups. On the contrary, some other studies have shown that curcumin supplementation has no effect on HSI and VSI, as detected in largemouth bass [72]. However, we were unable to compare our results with other relevant studies due to the lack of literature on the effects of NCur on the reproductive performance of farmed tilapia or other food fish and shrimp. Therefore, the current results will be discussed and compared with curcumin in its free form fed to other animals.

Pituitary gonadotropins (GtHs) such as luteinizing hormone (LH) and follicle-stimulating hormone [56] play a crucial role in regulating gametogenesis in teleost fish. LH is responsible for processes like milt and sperm production, ovulation, and oocyte development, while FSH controls early stages of gametogenesis such as sperm production and vitellogenesis [73]. 17 β-estradiol, an estrogenic steroid hormone, stimulates vitellogenin biosynthesis, leading to genital growth and female sexual maturation [74]. Testosterone, an androgenic steroid hormone, governs spermatogenesis and supports male reproductive functions [75]. Progesterone acts as a key steroidogenic mediator in spermatogenesis, sperm maturation, and oocyte growth and maturation in teleosts [76]. The secretion of gonadotrophic hormones serves as a vital marker for fish reproduction [76].

The results of the study showed that the sex hormone concentration of red tilapia fed diets supplemented with NCur significantly increased compared to fish fed the control diet, free Cur, or blended Cur. Similar to these findings, a study by [25] indicated that a diet containing 2.4–4.8 g of curcumin per kg of ration for Siam catfish (*Pangasianodon hypophthalmus*) could lead to higher plasma concentrations of 17β-Estradiol and vitellogenin. The present results clearly indicate that nano-curcumin can improve the reproductive endocrine function of red tilapia, leading to follicular maturation. Several studies have shown the positive effects of curcumin on the female reproductive system by positively impacting physiological processes such as the release of ovarian hormones, follicular development, puberty, and reproductive aging [77, 78]. For example, a study by [79] found that aging mice fed 100 mg of curcumin per kg of body weight showed improvements in indicators of ovarian reserve, such as FSH and estradiol serum levels. Additionally, [12] demonstrated that curcumin administration improved the serum levels of testosterone, LH, FSH, estradiol, and progesterone in female rats. Similarly, rabbits treated with curcumin showed increased production of testosterone and progesterone [77].

In the current study, other female reproductive parameters (GSI%, egg diameter and fry weight) followed the same hormonal pattern. This suggests that nano-curcumin can improve the reproductive capacity of this fish. According to [80], curcumin supplementation in feed can increase the egg diameter, gonadosomatic index and the mature sex percentage of red fin shark (*Epalzeorhynchos frenatus*) in its non-spawning season. Similar observations were reported in the follicle’s diameter of quail [81] and Magelang ducks [80]. At the same line, [70] demonstrated that the combination of curcumin and thyroxine in catfish diet increased its vitellogenin content, egg diameter, triglycerides concentration, relative fecundity, fertilization rate, hatching rate, and deposition of vitellogenin in the ovulated eggs, thereby improving the egg quality and reproductive performance of female broodstock. Moreover, an increase in egg diameters and hormone reproductive levels (estradiol and testosterone) of striped catfish (*Pangasionodon hypophthalmus)* fed a combination of curcumin, Pregnant Mare Serum Gonadotropin (PMSG) and anti-dopamine were detected outside its spawning season [71].

The histological results indicate that gonadal maturity is fastest in male and female red tilapia fed 60 mg NCur kg^−1^, followed by the fish group fed Cur/NCur and then the free Cur group, compared to the control fish group. This is evidenced by the increasing number of spermatozoa in the testes and developing oocytes in the ovaries of fish fed free NCur, with a higher incidence of oogonia and oocytes in different developmental stages. Red tilapia supplemented with 60 mg NCur kg^−1^ in their diet showed better gonadal maturity, indicating readiness for spermiation, ovulation, and spawning. These results are consistent with previous studies [82], which found that curcumin accelerates the process of gonad maturation. The hepatoprotective activity of curcumin improves the biological conditions of hepatic cells, leading to the synthesis of vitellogenin under the stimulation of 17 β-Estradiol. This enhances hepatic performance and metabolism of nutrients for follicular growth and maturation. Another study [71] reported that curcumin added to the diet of striped catfish accelerates gonadal maturation by histologically increasing the size of oocytes [69].

Furthermore, research [83], showed that curcumin improves the reproductive performance of catfish by increasing vitellogenin concentrations in eggs and accelerating gonadal development. The phytoestrogenic effect of flavonoids in curcumin stimulates vitellogenin synthesis, supporting fish reproduction. Cur also promotes steroidogenesis, folliculogenesis, and ovarian growth, as well as enhancing male hormones, sperm quality, and reducing reproductive toxicity. The enhancement of gonad maturity may be also referred to the phytoestrogenic effect of flavonoid, the biochemical composition of curcumin, which acts as estrogen to stimulate the synthesis of vitellogenin (the precursor of egg yolk from liver) supporting fish reproduction [80]. The current study demonstrates that NCur's hepatoprotective activity improves the reproductive performance of red tilapia and teleost fish. Curcumin's phytoestrogenic properties and ability to promote vitellogenin synthesis play a crucial role in enhancing gonadal maturity and reproductive functions in fish [83].

Previous studies have reported that curcumin promotes steroidogenesis, folliculogenesis, and ovarian growth [78]. This is attributed to the presence of phytoestrogens in curcumin, which have similar features to estradiol, as they are capable of inducing the biosynthesis of vitellogenin in fish liver [24]. Additionally, curcumin can enhance male hormones, sperm morphology and motility, sperm count, and reduce reproductive toxicity [56, 84]. Other reports have shown that curcumin directly increases apoptosis and reduces proliferation in ovarian cancer cells [85], and it can also protect against the harmful effects of oxidative stress on ovarian function [12].

Dietary Cur also plays a protective role in the male and female reproductive systems by improving gonadal function, growth, and reproduction. It regulates reproductive-related hormones such as testosterone, FSH, and LH serum levels, and promotes development at all spermatogenic and follicular stages in mice and rabbits [56, 77]. Cur can alleviate reproductive toxicity by supporting antioxidant enzymes, which help decrease oxidative stress and lipid production, maintaining steroidogenesis and spermatogenesis processes [84]. It also restores altered ovarian histology and follicular maturation deteriorations [86].

Reproduction-associated genes, such as CYP19A1A, FSHR, FOXL2A, ESR1, ESR2A, and pgr, play crucial roles in sex differentiation, gonad development, and reproductive efficiency in fish [87]. The aromatase gene *CYP19A1A* is essential for sex differentiation in both males and females and is involved in the metabolism of E2 in mammals and fish [88]. The *FSHR* gene functions as the receptor for follicle-stimulating hormone and plays a role in gonad development. *FOXL2A* is involved in ovarian development and function in various fish species, while progesterone regulates the formation of primordial follicles. Additionally, ESR1 is crucial for ovulation efficiency in females, and ESR2a facilitates the effects of 17α-ethinylestradiol on the distribution of primordial germ cells in fish [89].

The expression of these genes in red tilapia fed 60 mg NCur kg^−1^ diets in the present study was highly upregulated compared to fish fed the control diet. This is attributed to the antioxidant, immunostimulant, and hepatoprotective activities of NCur in fish. Based on the properties of curcumin, a study by [90] revealed its beneficial role in the testis and ovaries through various mechanisms, such as its effects on the expression profile and associated signaling pathways. These molecular results confirm the positive effects of nano-curcumin on fish reproduction and support the results presented on hematological and biochemical parameters, as well as reproductive performance (GSI, egg diameter, and larval production). In line with the current results, [24] postulated the positive effects of Cur supplementation in the diet of catfish, which improves gene expression, biological functions, and hormonal levels of fish broodstock, thereby enhancing the transfer of nutrients, gene products, and hormones to the developing oocytes. Reports by [79] also indicated that curcumin as herbal medicine and food additive can maintain the ovarian reserve through anti-inflammatory and endocrine regulation of the female reproductive system, as demonstrated by their ovarian in vivo and in vitro experiments and single-oocyte qPCR results. In contrast, [86] found no change in the gene expression of *FSHR* in the ovaries of Wistar rats exposed to formaldehyde and treated with curcumin. Due to the limited number of studies, it has been reported that phytoestrogens in plants like eurycomanone affect the expressions of reproductive-associated genes in fish. A significant increase in the expression of *CYP17A1, STAR, CYP19A1, SOX9A*, and *DMRT1* in the testis and an up-regulation of *CYP17A1, CYP19A1, 17B-HSD, STAR*, and *FTZ* genes in the ovary of *Clarias magur* fish, respectively injected with eurycomanone and chitosan-conjugated eurycomanone, were detected in the study of [91].

In the present study, the positive results of the Cu/NCur blend could potentially explain a synergistic effect of curcumin and its nano form in enhancing fish response. This may indicate the role of NCur in efficiently delivering free Cur to target sites in the fish body [32] as free Cur has metabolic limitations such as poor absorption, low availability, fast metabolism, and excretion [28]. Therefore, blending NCur with Cur in the current experiment could elevate the systemic levels of Cur by enhancing its absorption, biocompatibility, and biodegradability.

In the current study, it was found that a concentration of 60 mg NCur kg-1 achieved the best results in fish reproductive traits compared to fish fed free Cur or NCur/Cur blend. This concentration was able to regulate sexual hormone levels, enhance gonadal maturation stages, improve egg diameter, and up-regulate the expression of reproduction-associated genes. Similar positive effects of NCur have been reported in Nile tilapia [31, 32] and red tilapia [21]. Therefore, it is suggested that incorporating NCur in the diet of red tilapia broodstock is superior to using free curcumin or NCur/Cur blend for improving overall reproductive performance.

## Conclusion

In conclusion, the current study revealed that dietary administration of curcumin nanoparticles at a dosage of approximately 60 mg/kg is superior to free curcumin or a blend of NCur/Cur. This administration improved hemato-biochemical parameters, enhanced stages of gonadal maturation, and overall reproductive performance of red tilapia broodstock. Therefore, the study recommends the use of NCur in the diet of red tilapia over the traditional form to reduce feed costs and provide consumers with a healthier and more organic profile of raised aquaculture fish species.

## Data Availability

The data applied along with this investigation are available with the corresponding author upon reasonable request.

## Abbreviations

Cur: Curcumin
Cur/Ncur: Curcumin/nano-curcumin blend
Ncur: Nano-curcumin
TEM: Transmission electron microscope
CP: Crude protein
RBCs: Red blood cells
WBCs: White blood cells
MCV: Mean corpuscular volume
MCH: Mean corpuscular Hemoglobin
MCHC: Mean corpuscular hemoglobin concentration
Hb: Hemoglobin
PCV: Packed cell volume
AST: Aspartate aminotransferase
ALT: Alanine aminotransferase
ALP: Alkaline phosphatase
GSI: Gonadosomatic index
T: Testosterone
LH: Luteinizing hormone
E_2_: Estradiol
Prog: Progesterone
HSI: Hepatosomatic index
CYP19A1A: Cytochrome P450 family 19 subfamily A member 1
FSHR: Follicle-stimulating hormone receptor
LHR: Luteinizing hormone receptor
FOXL2A: Forkhead box L2
ESR1: Estrogen receptor 1
ESR2A: Estrogen receptor 2a
PGR: Progesterone receptor
